# The Treatment of Nonmelancholic Depression: When Antidepressants Fail, Does Psychotherapy Work?

**DOI:** 10.1177/070674371405900703

**Published:** 2014-07

**Authors:** Gordon Parker, Rebecca Graham, Elizabeth Sheppard

**Affiliations:** 1 Psychiatrist and Scientia Professor, Black Dog Institute, University of New South Wales, Sydney, Australia.; 2 Researcher, Black Dog Institute, University of New South Wales, Sydney, Australia.; 3 Clinical Psychologist, Black Dog Institute, Sydney, Australia.

## Abstract

**Objective:**

Treatment-resistant depression (TRD) is used as a descriptive or diagnostic term and has generated many management guidelines weighting antidepressant (AD) therapy, but which may be an inappropriate paradigm for the nonmelancholic disorders where psychotherapy may be a more salient modality. This study sought to evaluate the effectiveness of psychological therapy in patients whose nonmelancholic depressive condition had been resistant to at least 2 ADs.

**Method:**

Principal analyses compared 32 patients, diagnosed with a nonmelancholic depression who received 12 weeks of psychological therapy, with a small control group. Comparative analyses failed to find a distinct therapeutic effect, leading to an extension study pursuing candidate explanatory factors for this lack of response, including psychosocial factors.

**Results:**

While our sample showed a 41% response and 22% remission rate to psychotherapy, their improvement pattern was similar to the control group, thus arguing against any specific therapeutic benefit. Explanatory factors nominated by the treating psychologist weighted personality issues for 35% of the patients, distal stressors for 22%, and comorbid anxiety conditions for 18%. When sample members were compared with an age- and sex-matched sample of patients with nonmelancholic depression who improved distinctly during a similar 12-week period, rates of such putative personality, stress, and anxiety risk factors did not differ, arguing against the likelihood of these factors compromising improvement.

**Conclusions:**

Patients with nonmelancholic TRD also failed to demonstrate a clear response to a psychotherapeutic approach, while our pursuit of clinically explanatory variables was not supported empirically.

## Abstract (fr)

**Objectif:**

La dépression réfractaire au traitement (DRT) sert de terme descriptif ou diagnostique et a produit de nombreux guides de prise en charge pondérant la thérapie par antidépresseur (AD), mais peut être un paradigme inapproprié pour les troubles non mélancoliques, où la psychothérapie peut être une modalité plus indiquée. Cette étude cherchait à évaluer l’efficacité d’une thérapie psychologique chez des patients dont l’état dépressif non mélancolique avait résisté à au moins 2 AD.

**Méthode:**

Les analyses principales ont comparé 32 patients chez qui on a diagnostiqué une dépression non mélancolique et qui ont reçu une thérapie psychologique de 12 semaines, avec un petit groupe témoin. Les analyses comparatives n’ont pas constaté d’effet thérapeutique distinct, ce qui a mené à une prolongation de l’étude à la recherche des facteurs explicatifs de cette absence de réponse, y compris facteurs psychosociaux.

**Résultats:**

Même si notre échantillon affichait un taux de réponse de 41 % et un taux de rémission de 22 % pour la psychothérapie, leur modèle d’amélioration était semblable à celui du groupe témoin, ce qui allait donc à l’encontre de tout bénéfice thérapeutique spécifique. Les facteurs explicatifs suggérés par le psychologue traitant pondéraient les questions de personnalité pour 35 % des patients, les stresseurs distaux pour 22 %, et les troubles d’anxiété comorbides pour 18 %. Lorsque les membres de l’échantillon ont été comparés avec un échantillon de patients non mélancoliques appariés selon l’âge et le sexe qui s’étaient améliorés distinctement durant une période semblable de 12 semaines, les taux de ces facteurs éventuels de personnalité, de stress et de risque d’anxiété ne différaient pas, ce qui plaide contre la probabilité que ces facteurs compromettent l’amélioration.

**Conclusions:**

Les patients souffrant de DRT non mélancolique n’ont pas démontré non plus de réponse claire à une approche psychothérapeutique, alors que notre recherche de variables cliniquement explicatives n’a pas été soutenue empiriquement.

**Numéro d’enregistrement d’essai clinique :** ACTRN12611000932965

Treatment-resistant depression remains a clinical conundrum in terms of identifying underlying mechanisms and formulating management options. A recent review^1^ highlighted the keen interest in the topic illustrated by more than 2600 published articles reviewing TRD and related constructs during the preceding 5 years. Despite such a wealth of publications, Möller et al^1^ stated that there is neither an agreed on operational definition of TRD nor has any distinct phenotypic pattern been identified. TRD is sometimes positioned as synonymous with chronic depression, although the latter refers more to a prolonged and enduring depressive condition and the former weights failure to respond to multiple strategies and with minimal or no emphasis on its duration.^2^ Other overlapping terms consistently used are refractory depression, difficult to treat depression, and residual depression. Therefore, TRD may overlap conceptually and clinically with other terms, and there is no universally accepted definition.

Despite such ambiguities, TRD is a common clinical phenomenon. An early report^3^ quantified that one-third of depressed patients fail to respond to an initial AD, while an additional 50% partially respond. The more recent STAR*D study^4^ quantified remission rates across the first 4 treatment steps (with a complete description of the treatment steps provided in Rush et al^5^) of 37%, 31%, 14%, and 13%, respectively. STAR*D treatment steps principally involved differing ADs, although a percentage received CBT, either alone or in combination with an AD. If patients did not achieve remission or could not tolerate a treatment step, they were encouraged to proceed to the next acute treatment step. The cumulative remission rate after those 4 steps was 67%, indicating that one-third were nonremitters after that fourth stage—a nonremission rate consistent with the earlier estimate.^3^

While TRD is sometimes applied as a diagnosis, it may be better positioned as a descriptor—capturing treatment nonresponse across heterogeneous unipolar and bipolar depressive disorders and reflecting multiple possible determinants. Determinants may be biological (for example, rapid metabolizing status), psychological (for example, a personality contribution), social (for example, SLEs), or reflect the differential effectiveness and salience of drug and nondrug treatment modalities across constituent depressive conditions.

**Clinical Implications**

- Patients with a nonmelancholic depression resistant to ADs may also fail to improve with psychotherapy, arguing against treatment paradigm failure and more for treatment resistance.
- If such resistance does reflect personality-based factors and (or) SLEs, it may argue for a more extended psychotherapeutic treatment.

**Limitations**

- Sample numbers were low in our control group, with insufficient power, thus compromising identification of any significant differences.
- Subsidiary qualitative analyses were based on the psychologists’ nominations for depression persistence and hence subjective in nature.

An operational model was proposed by Thase and Rush^6^ that defines TRD by weighting response to ADs, and where they detailed a 4-item staging model, progressing across nonresponse from no medication having been tried; an adequate trial of 1 or 2 differing and adequate ADs; failure to respond to 2 differing augmentation strategies; and failure to respond to ECT. Such a model has strongly influenced the conceptualization and management of TRD, with the now commonly accepted definition being failure to respond to 2 or more AD trials of adequate dose and duration.^7^ Such a definition weights resistance only in relation to physical treatments, such as ADs and ECT, and effectively ignores causal or diagnostic factors. In fact, psychotherapy is generally positioned as an adjunct treatment rather than a primary treatment option for TRD. Despite some indication from a small RCT that CBT may be more effective for TRD than TAU (see Wiles et al^8^ for more details), recent reviews^7^–^9^ highlight the paucity of RCTs examining TRD and CBT, and with results to date indicating no additional benefit of CBT over medication.

While these meta-analyses highlight the efficacy of ADs in the management of MDD others have established the comparable efficacy of psychotherapeutic approaches—in particular, the evidence-based therapies of CBT and IPT^10^—again in managing MDD. Currently, there has been no definitive study identifying characteristics of patients with MDD who might respond preferentially to either medication, psychotherapy, or to another salient treatment approach. Instead, the choice of one treatment modality over another is likely to reflect clinical judgment, professional discipline (for example, primary practitioner, psychiatrist, or psychologist), and training factors rather than any empirical literature.

Our model of MDD is that it is a domain diagnosis capturing heterogeneous constituent conditions that may reflect primary biological, social, or psychological etiological factors. Theoretically, it may be expected that selection of the treatment modality may marry with and seek to address the principal cause. A quintessential biological condition is melancholic depression, where its treatment ascriptions include a preferential response to physical treatments, such as drugs and ECT, a poorer response to psychotherapy, and a low placebo response.^11^ By contrast, CBT and IPT assume an etiological predisposition emerging from the individual’s personality style and psychosocial stressors, respectively, and weight interventions targeting such causal factors. If, in fact, TRD emerges from personality-based predispositions or SLE precipitants, the role of ADs may be limited and effectively create a (false-positive) TRD scenario. In such situations, failure to respond may not reflect treatment resistance—but more a paradigm failure, with treatment choice not matched to causal factors.^12^ Our model of the nonmelancholic depressive disorders is that they are principally a consequence of antecedent stressors and (or) a vulnerable personality style, as detailed by Parker and Manicavasagar.^10^ Assuming that a diagnosis of TRD in people with a nonmelancholic depression may reflect failure to provide a cogent nondrug therapy designed to address these factors, we designed a study to determine the impact of a paradigm change to management. We report key study analyses and undertook an extension study to determine possible explanatory factors.

## Methods

### Intervention Study and Control Groups

We sought to recruit patients receiving a clinical diagnosis of a nonmelancholic depression who had failed to report any distinct improvement to 2 or more adequately trialled ADs during their lifetime and who had not received psychological treatment in the preceding 3 months. Eligible patients (treatment group) were invited to receive psychotherapy from an experienced clinical psychologist. People unable to attend weekly treatment sessions owing to geographical reasons were assigned to a control group.

### Recruitment and Assessment

The sample was derived during the 2009 to 2012 period from patients (over 18 years) attending the BDI Depression Clinic, a state-wide tertiary service for patients with a primary mood disorder. Clinic patients were referred by their managing doctor for diagnostic and management advice in relation to persisting and (or) severe depressive conditions.

All patients received a detailed clinical assessment by a BDI psychiatrist. A clinical diagnosis of nonmelancholic depression required the absence of any prototypic^13^ melancholic features (for example, psychomotor disturbance, a nonreactive and anhedonic mood, anergia, and diurnal variation of mood and energy) and the presence of putative nonmelancholic features, such as mood reactivity and increased appetite and (or) food cravings. Study exclusions were excessive drug or alcohol use, a primary medical problem or contributory organic condition, or a substantive alternative primary diagnosis. We viewed the psychotherapeutic intervention as primary (and under evaluation), and therefore did not exclude patients if their managing therapist changed their medication and (or) dosage during the study course.

Prior to clinical assessment, patients completed the computerized Mood Assessment Program, which generates sociodemographic and clinical information, including age at depression onset, current length of depressive episode, medical history, number of previous medications trialled, impact of current and SLEs, family history of mood disorders, history of anxiety disorders or use of illicit drugs and alcohol, medications (past and present), organic and medical conditions, and personality styles. The last was assessed by the T & P Questionnaire,^14^ which generates 10 scale scores, with 8 (that is, social avoidance, irritability, perfectionism, anxious worrying, personal reserve, self-criticism, interpersonal sensitivity, and self-focused) assessing at-risk temperament and personality styles to depression and 2 (that is, cooperativeness and effectiveness) assessing disordered personality function. The measure is psychometrically sound, with high internal consistency, test–retest reliability, and concurrent validity.^14^ All patients provided informed consent and the study was approved by the University of New South Wales Ethics Committee.

We aimed to quantify 12-week progress between people who accepted the psychotherapeutic intervention and those in an adequately sized TAU comparison group who were unable to attend the clinic for various reasons. Recruitment proved extremely difficult, and, during the 3-year period, only 41 patients met both eligibility criteria and consented to be in the treatment or control group. Therefore, we elected to cease recruitment after enrolment and 12-week completion of at least 30 patients, and with our final treatment sample totalling 32 patients, while our TAU control group had only 8 patients. Despite limited comparison sample sizes, analyses (as shown below) allow interpretation of progress in the treatment group to be offered with some degree of confidence.

### Primary and Secondary Study Measures

A battery of questionnaires were completed by participants in both groups at study commencement (baseline), weekly intervals, and at 12-week study completion. The measures completed at these 2 time points are outlined in the following section.

#### Weekly Measures

Our primary measure was the 16-item, self-report version of the QIDS-SR,^15^ measuring depression severity. The QIDSSR has been shown to be sensitive to change to medications and psychotherapy treatments, and has adequate reliability and validity scores.^16^ Secondary weekly measures included the 14-item anxiety subscale of the Depression Anxiety Stress Scales^17^ and the 6-item Work Productivity and Activity Impairment Questionnaire,^18^ measuring work and activity impairment—with both measures having sound psychometric properties.^18^,^19^ Life enjoyment and satisfaction was measured by the 16-item Quality of Life Enjoyment and Satisfaction Questionnaire,^20^ which has been shown to have acceptable reliability and validity in the development study.

#### Measures Completed at Baseline and 12-Week Study Completion

In addition to the weekly measures, all patients completed 3 additional measures at baseline and 12-week study completion: the 54-item, self-report version of the SASSR,^21^ the anxiety scale of the CCDAS,^22^ and a clinic-developed, 6-item Overall Functioning Assessment (based on the Work and Social Adjustment Scale^23^), which measures global impairment during the preceding month. The SAS-SR is a widely used measure of social adjustment, with various studies attesting to its acceptable psychometric properties.^24^,^25^ The CCDAS is a measure of trait anxiety and has been shown to have acceptable reliability and concurrent validity.^26^ All measures were computer-completed, with the control group receiving an email with a link to the questionnaires at weekly intervals. Patients also underwent a structured diagnostic interview administered by a trained research assistant, involving mood disorder modules and the psychotic disorders section of the Mini International Neuropsychiatric Interview,^27^ to ascertain the presence of a major depressive episode at baseline and at study completion.

#### Therapeutic Intervention

Patients receiving psychological treatment from our BDI Depression Clinic psychologist received 10 sessions conducted during a 12-week period—the first 8 were weekly sessions, followed by 2 sessions conducted 2 weeks apart. The BDI psychologist had a formal clinical psychology degree and 10 years’ experience in providing psychological therapies to patients with depression. The treatment model was broadly based on our theoretical formulation of etiological and maintaining factors contributing to nonmelancholic depression, but was tailored to meet the particular needs of each patient. The treatment plan had some consistent components, including an initial focus on psychoeducation about nonmelancholic depression, the identification of short-and long-term stressors, and an examination of coping styles. Subsequent sessions focused more on individual personality characteristics, cognitions, and schemas that were formulated as contributing to the persistence of the patient’s depression. Although a range of psychological approaches were used, the main ones were CBT (*n* = 27), IPT (*n* = 8), schema therapy (*n* = 5), acceptance and commitment therapy (*n* = 2), dialectical behavioural therapy (*n* = 2), and mindfulness training (*n* = 2).

### Statistical Analysis

Principal analyses involved independent Student *t* test and chi-squared analyses of all study variables compared from baseline to the final therapeutic session after 12 weeks. Data management and analyses were conducted using SPSS, version 20.0 (SPSS Inc, Chicago, IL).

## Results

### Comparison of Principal Study Groups

The treatment (*n* = 32) and control (*n* = 8) groups did not differ by mean age (mean 43.2, SD 13.7 and mean 49.0, SD 8.3, respectively; *t* = 1.07, *df* = 38, *P* = 0.29), marital status (χ^2^ = 0.98, *df* = 2, *P* = 0.61 across 3 categories), employment status (χ^2^ = 5.31, *df* = 4, *P* = 0.26 across 5 categories), or education level (χ^2^ = 2.54, *df* = 4, *P* = 0.64 across 5 categories). The female preponderance was lower in the treatment group (51.5%) than the control group (62.5%) but nonsignificant (χ^2^ = 0.31, *df* = 1, *P* = 0.58). The treatment and control groups reported a similar age of depression onset (21.0 and 25.0 years), and current depressive severity (QIDS-SR = 15.6 and 14.6), although the treatment group reported a nonsignificantly shorter current depressive episode (74.4 and 81.4 days). The treatment group had previously trialled (over their lifetime) fewer ADs for their depression than the control group (3.6 and 5.9, respectively; *t* = 3.06, *df* = 38, *P* = 0.004).

Table 1 reports baseline and study completion data for the patients receiving treatment. There was a significant improvement on our primary measure of depression severity (the QIDS-SR) and on all secondary measures other than the work productivity and quality of life measures, and the trait anxiety subscale. The responder rate (that is, at least a 50% reduction in QIDS-SR scores) was 40.6%, while remission (that is, a QIDS-SR score of 5 or less) was achieved by 21.9%.

**Figure 1:**
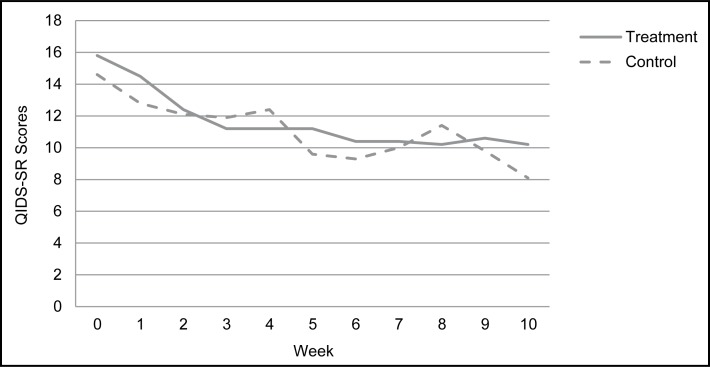
Distribution of weekly depression severity as measured by the Quick Inventory of Depressive Symptomatology—Self-Report (QIDS-SR) for the treatment and control study groups

Table 2 reports change scores on all study measures by patients in the treatment and control groups, with analyses failing to quantify any significant differences on any study measure. While patient numbers in the control group were low, the parallel changes in study measure scores across the 2 groups were quite striking. As an illustration, we plot only weekly scores on our primary QIDS-SR measure (Figure 1). Seemingly identical trajectories of improvement in QIDSSR scores are evident in both groups. This suggests that any improvement in people receiving psychotherapy likely reflects a natural remission or related phenomenon rather than any specific therapeutic effect. Thus our hypothesis that provision of an active psychotherapeutic intervention would be a more salient and effective treatment modality in patients with a nonmelancholic TRD was not supported. Subsequent analyses explored possible explanations.

### Qualitative Analyses

We invited our study psychologist to provide a formulation for treatment group participants of contributory factors, to both onset and depression persistence, as identified at initial assessment and during the therapy course. The psychologist was required to select the relative contribution for each patient (from 5 a priori options and with multiple options allowed) of the explanatory factors judged as potentially compromising any distinct therapeutic response—whether or not the patient actually improved across the intervention period. The most commonly nominated factor (mean 35%, SD 13.7) was the patient’s personality style (for example, rejection sensitivity, self-criticism, and anxious worrying) or the presence of a personality disorder (for example, avoidant, narcissistic, and borderline). Next (mean 22%, SD 12.9) was the impact of distal childhood stressors, such as childhood sexual abuse or absent and (or) limited parental attachment bonds. Other commonly selected factors included the presence of a comorbid anxiety condition (mean 18%, SD 11.4), enduring stressors, such as financial or employment issues (mean 12%, SD 9.6), and recent stressors, such as a marital breakdown or the loss or death of a significant other (mean 12%, SD 12.7).

In relation to depression persistence, personality style or personality disorder nuances were nominated for 18 patients (55%). Other factors included the continuing impact of distal stressors, such as childhood abuse (*n* = 8 or 24%), comorbid factors, such as anxiety (*n* = 4 or 12%), recent severe stressors (*n* = 2 or 6%), and enduring or chronic stressors (*n* =1 or 3%).

### Subsidiary Quantitative Analyses

We subsequently undertook a quantitative study to examine the potential salience of the factors identified in the qualitative analyses to compromising response to therapy. Such an objective required a comparison with a patient sample, whose members had, by contrast, shown a substantive therapeutic response. Therefore, we derived an adequately sized age- and sex-matched group of independent patients (improver sample) diagnosed with a nonmelancholic depression at the BDI Depression Clinic and reported a 50% or more improvement on our primary study measure of depression severity (the QIDS-SR) at 12-week review—and with group inclusion, irrespective of the receipt of any new or modified (drug or nondrug) treatment regime. We hypothesized that our treatment sample would have higher rates of predisposing personality styles, anxiety conditions, and distal and proximal stressors than those in the improver sample.

The treatment (*n* = 32) and improver (*n* = 53) samples did not differ by mean age (43 years, SD 13.9 and 41 years, SD 12.6, respectively; *t* = 0.44, *df* = 82, *P* = 0.40), female preponderance (52% and 53%, respectively), marital status (χ^2^ = 2.42 *df* = 2, *P* = 0.30 across 3 categories), employment status (χ^2^ = 4.24, *df* = 4, *P* = 0.37 across 5 categories), or education level (χ^2^ = 1.73, *df* = 4, *P* = 0.78 across 5 categories)—suggesting adequate matching. The 2 groups did not differ by age at their initial depressive episode (21 and 22 years, respectively), but the treatment group had experienced a slightly (nonsignificant) longer current depressive episode (74 and 67 weeks, respectively). People in the treatment and improver groups had previously received a comparable number of ADs (3.6 and 3.2, respectively), but our treatment group tended to be more likely to have received 5 or more ADs (38% and 22%, respectively). They did not differ in number of current or past significant medical conditions (3.1 and 2.2, respectively), presence of anxiety disorders (generalized anxiety, social phobia, panic disorder, and obsessive– compulsive disorder), current number of stressors (3.0 and 2.7, respectively), rejecting parents (50% and 46%, respectively), and a family history of alcoholism (40.6% and 40.0%, respectively). However, the treatment group were more likely (than the improver group) to report a greater number of SLEs (5.5 and 3.8, respectively; *t* = 2.4, *df* = 832, *P* < 0.02) and did show a nonsignificant trend to be more likely to report a family history of depression (72% and 58%, respectively). On all T & P Questionnaire items, the groups returned comparable mean scores and without any indicative trend differences. Thus analyses suggested that people in the treatment group had experienced a somewhat longer current depressive episode and were somewhat more likely to have received at least 5 ADs, but did not differ on personality, anxiety, and stress constructs that we positioned as accounting for their TRD.

## Discussion

### Study Overview

We postulated that people with a nonmelancholic TRD who had received several ADs (quantified mean 3.6, SD 1.6) may not reflect true treatment resistance but more of a paradigm failure, and where a psychotherapeutic intervention addressing psychosocial determinants would be salient. If confirmed, this may lead to broader models for conceptualizing TRD and management algorithms for separate melancholic and nonmelancholic depressive disorders.

## Main Findings

The provision of psychotherapy by a highly trained psychologist to our sample of patients with nonmelancholic TRD was encouraging of our hypothesis, in that we quantified a 41% responder and a 22% remission rate on our primary measure of depressive severity. However, and despite having few control patients, the trajectory in weekly QIDS-SR scores in the treatment and control groups were strikingly similar, and therefore argued against a true therapeutic effect and more for a nonspecific therapeutic or even a nontherapeutic contribution to improvement (for example, regression to the mean and vicissitudes in life improving). The suggested failure of our treatment group to evidence any true therapeutic responsiveness argues for their having a depressive state resistant to both medication and psychotherapy and inviting the question as to why. Our qualitative analyses indicated numerous plausible candidates—such as the presence of a significant personality style or disorder, exposure to severe distal and (or) recent stressors, and the presence of a comorbid anxiety condition. However, the prevalence of such factors did not differ across our sample and a comparison sample that had shown substantive improvement during a similar 12-week interval.

## Study Limitations

Our analyses again argue the need (if not the necessity) for therapeutic studies having appropriate comparison or control groups, and we acknowledge that the small sample sizes (particularly in the control group) leads to a need to interpret these results with caution. In their absence, we might have argued that psychotherapy was a more cogent treatment model in that recipients showed a modest response to psychotherapy and imputed key determinants of treatment resistance (for example, personality style, stress, and anxiety) in our patients with nonmelancholic TRD. Our comparison groups effectively disallowed either conclusion to be made. In addition, while a study aim was to examine the effectiveness of changing the paradigm from a pharmacological to a psychological approach, most patients remained on ADs, which may have confounded study results, despite their suggested resistance to such medication. Subsidiary analyses were based on the treating psychologists’ nominations of reasons for treatment persistence, which, being subjectively based, may or may not have been valid in their identification and weighting.

## Conclusions

Sample numbers suggest that our study is best viewed as a pilot one, while findings invite more questions than provide clarity. If nonmelancholic depression is largely a consequence of debilitating SLEs interacting with a vulnerable and predisposing personality style, then it remains unclear as to why a percentage of people are resistant to drug and nondrug therapies, and particularly to the psychotherapy provided to our sample, which was designed to specifically address these factors. Possible explanations include the short therapy duration (in that such patients may need a longer period of psychotherapy to establish a therapeutic alliance and [or] obtain benefit), or a need for another paradigm rather than psychotherapy. In the introduction, we noted that TRD is a conundrum. Most treatment studies have focused on biological explanations and drug treatments. Our study explored the effectiveness of psychological therapy in a group of patients diagnosed with a nonmelancholic depression and examined whether psychotherapy was effective when previous AD treatments were not. Our results appear to broaden, rather than narrow, the conundrum, but suggest the need for future studies exploring a wide set of possible explanatory factors.

## Figures and Tables

**Table 1:** Paired Student *t* test comparing outcome measures at baseline and 12-week study completion for the treatment sample

Outcome measure	Baseline Mean score (SD)	Follow-up Mean score (SD)	*t*^a^	*df*	*P*
Quick Inventory of Depressive Symptomatology—Self-Report	14.9 (3.8)	9.3 (5.3)	5.61	32	<0.001
Depression and Anxiety Stress Scales	10.4 (8.0)	7.2 (7.7)	2.20	31	0.05
Work Productivity and Activity Impairment Questionnaire	85.5 (30.3)	96.8 (8.8)	1.36	15	0.19
Quality of Life Enjoyment and Satisfaction Questionnaire	37.6 (15.6)	43.8 (18.2)	1.95	32	0.06
Social Adjustment Scale—Self-Report	2.5 (0.5)	2.2 (0.5)	2.27	31	0.045
Costello and Comrey Anxiety Scale	39.4 (12.0)	39.3 (10.2)	0.07	28	0.95
Overall Functioning Assessment	14.6 (4.4)	1.4 (5.6)	3.10	31	0.005

a Student *t* tests were 2-tailed

**Table 2:** Independent Student *t* test comparing treatment and control groups on improvements on measures over time

Outcome measure	Treatment Mean score (SD)	Control Mean score (SD)	*t*^a^	*df*	*P*
Quick Inventory of Depressive Symptomatology—Self-Report	5.4 (5.4)	6.5 (7.3)	0.46	31	0.65
Depression and Anxiety Stress Scales	1.8 (7.1)	7.5 (10.8)	1.74	31	0.09
Work Productivity and Activity Impairment Questionnaire	14.4 (39.5)	4.2 (9.4)	0.56	14	0.58
Quality of Life Enjoyment and Satisfaction Questionnaire	4.6 (18.2)	11.2 (18.4)	0.89	31	0.38
Social Adjustment Scale—Self-Report	0.2 (0.6)	0.2 (0.5)	0.08	30	0.94
Costello and Comrey Depression and Anxiety Scale	−0.3 (6.1)	1.1 (3.4)	0.58	27	0.57
Overall Functioning Assessment	2.1 (4.7)	6.4 (7.7)	1.90	30	0.07

a Student *t* tests were 2-tailed

## Abbreviations

AD: antidepressant
BDI: Black Dog Institute
CBT: cognitive-behavioural therapy
CCDAS: Costello and Comrey Depression and Anxiety Scale
ECT: electroconvulsive therapy
IPT: interpersonal therapy
MDD: major depressive disorder
QIDS-SR: Quick Inventory of Depressive Symptomatology—Self-Report
RCT: randomized controlled trial
SAS-SR: Social Adjustment Scale—Self-Report
SLE: stressful life event
STAR*D: Sequenced Treatment Alternatives to Relieve Depression
T & P: Temperament and Personality
TAU: treatment as usual
TRD: treatment-resistant depression
